# Evaluation of compost quality from municipal solid waste integrated with organic additive in Mizan–Aman town, Southwest Ethiopia

**DOI:** 10.1186/s13065-021-00770-1

**Published:** 2021-07-19

**Authors:** Masresha Mamo, Henok Kassa, Lalit Ingale, Stefaan Dondeyne

**Affiliations:** 1 Department of Natural Resources Management, Mizan-Tepi University, PO. Box 391, Mizan Teferi, Ethiopia; 2 Department of Geography, Ghent University, Krijgslaan 281 S8, 9000 Gent, Belgium

**Keywords:** Municipal solid waste, Organic additives, Composting, Physico-chemical characteristics, Phytotoxicity

## Abstract

**Background:**

The present study evaluated the compost quality from municipal solid waste (MSW) and organic additives of coffee by-products and leaf of *Millettia ferruginea*. Compost sample (n = 30) was taken from fresh compost materials and MSW and different organic additive treatments (T1, T2, T3, T4, and T5). Compost treatments phytotoxicity test was conducted using lettuce seed (*Lactuca Sativa* L. var. crispa). Analysis of variance (ANOVA) was performed using SPSS (version 22) on major compost quality characteristics.

**Results:**

The compost Physico-chemical characteristics like temperature (26.4 °C), moisture content (45.5%), electrical conductivity (4.6 mS/cm), pH (7.9), total nitrogen (1.2%) and phosphorous content (2918 ppm) in T4 and T5 were analogous but both are significantly different from T3, T2 and T1 compost treatments. Phytotoxicity test using 100% compost treatment media showed that T4 (101%) and T5 (102%) are phytonutrient for lettuce plant. While, T3 and T2; and T1 compost treatments are non-phytotoxic and moderately phytotoxic respectively to lettuce plant.

**Conclusion:**

Therefore, compost from MSW + *M. ferruginea* (T4) and MSW + coffee pulp + *M. ferruginea* (T5) are important for improving the physico-chemical characteristics of compost and are phytonutrient for lettuce plant. Thus, for effectively management of the 75% of organic fraction of waste generated from households in the study area, recycling methods like composting with organic additives must be used at large.

**Supplementary Information:**

The online version contains supplementary material available at 10.1186/s13065-021-00770-1.

## Introduction

Municipal solid wastes are one of the global environmental challenges and major source of environmental pollution [1]. The increases in population, per capital income and expansion of urbanization have led to increase in variety and amount of municipal solid wastes [2]. Globally, over 2.0 billion tons of solid waste generated in 2016, with an average generation rate of 0.74 kg/person/day [2]. Similarly, Sub-Saharan Africa alone generated 174 million tons of waste in 2016 [3]. In Ethiopia, 6,532,787 tons of MSW generated per year in 2015 [4] and Addis Ababa alone generated a total of 754,236 tons of MSW per year, with an average per capital waste generation of 0.45 kg/person/day in 2018 [5].

In Ethiopian cities, municipal solid waste is managed using simple and cheapest option like disposing in landfills by means of open dumping [5]. This has been found to be the main source of urban pollution and potential threat to the public health, ecosystem and quality of life [6]. The generated waste composed of organic (60%), recyclable (15%) and others (25%) [7]. The municipal solid waste generation and management has been a major challenge for Bench Maji Zone towns, especially Mizan–Aman town. Solid wastes generation rate at household level in Mizan and Aman was 0.45 kg/capita/day in 2017, and the wastes are mainly composed of organic degradable waste (75%), plastic (10%), and mixed waste (15%). Municipal solid wastes were mainly disposed of as landfill in and around the towns, at road periphery and around the main river [8]. Poor municipal solid waste management practices have led to environmental quality deterioration like water, soil and air pollution, and human and livestock’s health hazards [6, 9].

Coffee by-products are potential threats for environmental quality and human health in coffee growing regions of Southwest Ethiopia, Bench Sheko Zone in particular. For example, every 100 kg of fresh coffee berry gives about 40 kg of wet coffee pulp waste [10, 11]. Poor disposal of coffee by-products like dumpling in landfill and water body have led to water and soil pollution [11]. However, utilization of coffee by-products by composting plays a critical role for organic fertilizer production and environmental protection [12]. Amending coffee by-product compost with organic materials like leaves of *Millettia ferruginea* (Hochst.) Baker and/or cow dung improve the quality of compost [12]. Composting using aerobic windrows method results a product that can be used as soil conditioner. Compost is a good organic fertilizer because it contains plant nutrients (N, P, K, Cu, Fe and Zn) as well as organic matter which improves its soil–water holding capacity and soil aeration, and thereby improve germinating seeds and plant root growth [12, 13].

Further, organic waste additives are important to facilitate the microbial enzymatic activity, shorten composting period, enhance the quality of matured compost and reduce pollution [14]. Southwest Ethiopia is known for its high coffee production, export, and disposable coffee by-products (coffee pulp, husk, and effluents), yet these wastes are disposed in landfill or openly incinerated, and thereby causing water and air pollution and human and livestock’s health risks [11, 12].

Currently, compost from municipal solid waste and other organic wastes have been practiced in various parts of globe and showed significant result in waste recovery and soil fertility amelioration [12, 15, 16]. Although, municipal solid waste and coffee by-products are potential threats for environmental quality and human health in the study area, yet little has been done on waste recovery and utilization by composting. Therefore, this study investigated the quality of compost from municipal solid waste and other organic additives in Bench Sheko Zone, Southwest Ethiopia.

## Materials and methods

### Study area

**Fig. 1:**
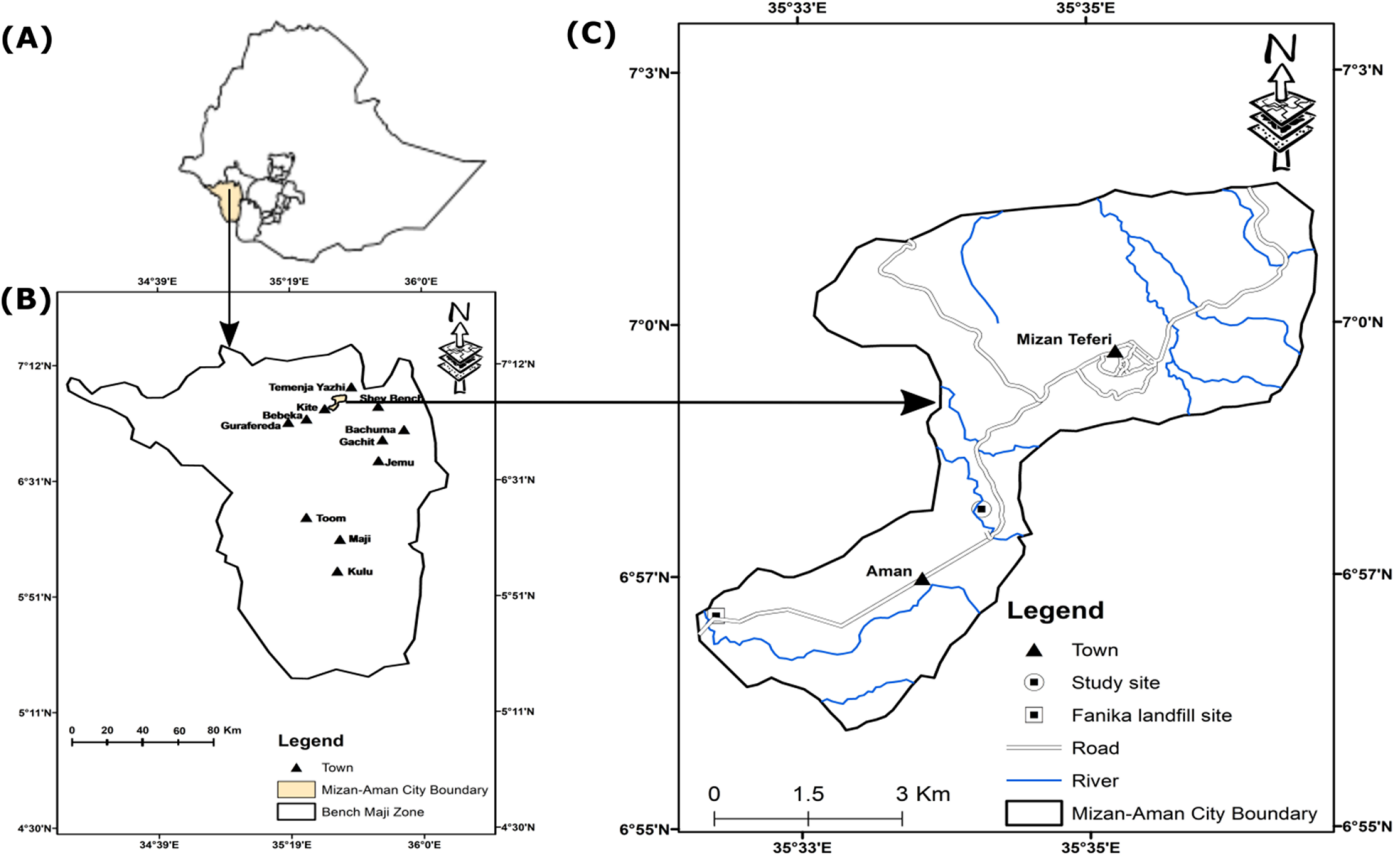
Study area: **A** location of study site in Ethiopia; **B** Mizan–Aman city boundary in Bench Sheko Zone; **C** location of study sites in Mizan–Aman city boundary, Southwest Ethiopia

This study was conducted in the tree nursery site at Mizan-Tepi University, Southwest Ethiopia (Fig. 1, Additional file 1). The university is located 555 km south west of Addis Ababa, the capital city of Ethiopia and 7 km far away from Mizan Teferi, the capital of Bench Sheko Zone. The study site is geographically located at 06° 57ʹ N and 035° 31ʹ E. The elevation of study area is 1276 m above sea level. In 2019, the total population of the town was 79,581, of which 40,678 were male and 38,903 were female [17]. The average annual rainfall distribution varying from 1801–2000 mm and the average air temperature ranges from 13 to 27 °C [18].

Fanika solid waste landfill site is located at the lower elevation of Gacheb catchment (Fig. 1). Gacheb catchment is a source of ‘Eseni’ and Gacheb River drains toward White Nile Basin. Fanika landfill site was established in 2015, mainly for dumping of the solid wastes generated from Mizan–Aman community and the surrounding kebeles. Based on the daily MSW generation rate (0.45 kg/person/day) [7] and total population (79,581) of the study area [17], the daily solid waste generation is expected to be 35,811 kg per day.

### Collection and processing of composting materials

The main organic materials for composting are municipal solid waste, coffee by-products and leaves of *M. ferruginea* (Hochst) Baker. Municipal solid waste was collected from Mizan–Aman town residential, hotel and market waste landfill sites. Coffee pulp and effluent was collected from nearby wet coffee processing station. *M. ferruginea* leaves and topsoil were collected from Fanika participatory forest site around Aman town. The collected municipal solid wastes were separated manually using hand into organic, plastics, metals, and inert materials. The MSW composition consists of 80% organic, 10% plastic and 10 others. All organic MSW and leaves of *M. ferruginea* composting materials were chopped manually into small pieces to aid the microbial activity [19].

### Composting experiment layout and pile setup

The composting experiment was done in Mizan-Tepi University at the tree nursery site in July 2019, under shade condition for 70 days. Compost samples were collected from four different points (top, center, side, and bottom) and those sub-samples were mixed properly for Physico-chemical analysis. All the compost treatments temperature was measured daily using portable compost temperature probe (Reotemp FG20P) at different points (top, center and middle). All compost treatments samples were sealed in plastic bag and transported to National Soil Testing Center, Addis Ababa for analysis. All the study samples were analyzed using the standard analytical procedures (Additional file 2).

The compost treatments proportion with additives on dry weight basis was as follows:

$$ {\text{Treatment}}\;{1}\left( {{\text{T1}}} \right){\text{:MSW}}\left( {{9}0{\text{ kg}}} \right) + {\text{topsoil}}\left( {{3}0{\text{ kg}}} \right), $$

$$ \begin{aligned} & {\text{Treatment 2}}\left( {{\text{T2}}} \right){\text{:MSW}}\left( {{9}0{\text{ kg}}} \right)\\ &\quad + {\text{coffee effluent}}\left( {{2}0{\text{ kg}} + {\text{topsoil 1}}0{\text{ kg}}} \right), \end{aligned} $$

$$ \begin{aligned} & {\text{Treatment 3}}\left( {{\text{T3}}} \right){\text{:MSW}}\left( {{9}0{\text{ kg}}} \right)\\ &\quad + {\text{coffee pulp}}\left( {{2}0{\text{ kg}}} \right) + {\text{topsoil}}\left( {{1}0{\text{ kg}}} \right), \end{aligned} $$

$$ \begin{aligned} & {\text{Treatment }}4\left( {{\text{T}}4} \right){\text{:MSW}}\left( {90{\text{ kg}}} \right) \, \\ &\quad + Millettia \, ferruginea{\text{ leaves}}\left( {20{\text{ kg}}} \right) \\ &\quad + {\text{topsoil }}10{\text{ kg}}, \end{aligned} $$

$${\text{Treatment 5}}\left( {{\text{T5}}} \right){\text{:MSW}}\left( {{9}0{\text{ kg}}} \right) + {\text{coffee pulp}}\left( {{1}0{\text{ kg}}} \right) + Millettia \, ferruginea{\text{ leaves}}({1}0{\text{kg}}) + {\text{topsoil}}\left( {{1}0{\text{ kg}}} \right).$$

**Fig. 2:**
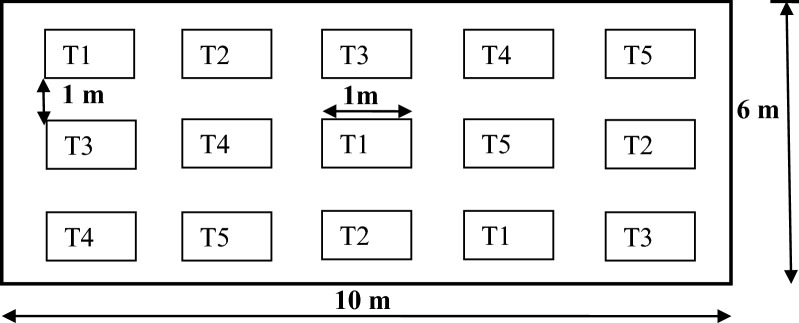
Experimental Layout

A report showed that leaves of *M. ferruginea* with 10–20 kg and coffee by-products (20–60 kg) proportion in the composting material is important to produce quality compost [12, 16]. The compost experiment was laid in a randomized complete block design, with three replicates (Fig. 2). All composting treatments were piled on heap of 1m^3^ size (1 m × 1 m × 1 m; length, width, and height), based on pile recommendation for wet region [20]. All the treatments piles were manually turned using shovel and rake once a week on the first 7 weeks of composting and once on every 2 weeks afterward. The piles of each composting materials were manually mixed using shovels during compost turning [21]. Compost piles moisture content was monitored regularly, and water was uniformly supplied on regular basis. The experiment treatments consist of the municipal solid waste alone as control and MSW with others organic additives as supplement for compost quality amendment. Topsoil was added for all the treatments to facilitate the decomposition process.

### Compost physico-chemical analysis

The compost moisture content was determined as weight loss upon drying in an oven dry (Henan Lanphan DZF6050) at 105 °C to a constant weight [22]. Bulk density was measured using an approximately 10 L volume container. The container was filled with material, and then the material was slightly compacted to ensure absence of large void spaces. The bulk density was calculated by dividing the weight of the material by the volume of material in the container [23]. Compost pH was measured using pH meter (Hanna HI5222). Electrical conductivity was measured from compost-water (1:2.5) suspension and in the supernatant, using conductivity meter (Jenway 4510) [24]. Organic matter content of the compost was determined by measuring the loss of ignition at 550 °C for 4 h using muffle furnace (ThermolyneF-6125M). The organic carbon (OC) was calculated using the following equation:

1 $$ {\text{OC }}\left( \% \right) = \frac{{\% {\text{ OM}}}}{1.724} $$

where OC is the organic carbon, OM is the organic matter, 1.724: is Van Bemmelen factor commonly used for organic carbon determination, based on the assumption that humified organic matter of soil contain about 58% organic carbon.

Total nitrogen (TN) was analyzed by Kjeldahl digestion method [23]. Total phosphorous was determined using spectrophotometer (Jenway 6320D) [23].

### Compost phytotoxicity test

The matured compost phytotoxicity was determined using seeds of lettuce (*Lactuca sativa* L.) var. crispa in Mizan Tepi University Greenhouse. The experiment was carried out based on complete randomized design (CRD) with three replicates. Soil and compost composite (3:1) and compost (100%) media proportion was used for phytotoxicity test in 5-L plastic pot. Lettuce was selected because of its sensitivity to the presence of phenolic compounds in the growth media and seed germination was inhibited by immature compost [25]. The germination test was carried out using 10 seeds of lettuce (*Lactuca sativa* L.) var. crispa then at the end of the 2 weeks; lettuce seeds germination and root length were recorded. The variety was selected because of its adaptive to southwest Ethiopia [26]. The germination index (GI) was determined considering the number of sprouts and root growth, using the following equation:

2 $$ {\text{GI}} \left( \% \right) = \frac{{{\text{GB}}}}{{{\text{GT}}}}*\frac{{{\text{LB}}}}{{{\text{LT}}}}*100 $$

where GB is the number of germinated seeds in a compost media pot, GT is the number of germinated seeds in a control compost media pot, LB is the root length on compost extract, and LT is the root length control.

Germination index is an integral parameter, which combines the relative germination and relative root elongation. A study reported that root elongation is more sensitive to the presence of toxin than seed germination [27].

A germination index values below 50% indicate high phytotoxicity; value between 50 and 80% indicate moderate phytotoxicity; and values above 80% indicate the absence of phytotoxicity. When the index exceeds 100%, the compost can be considered as a phytonutrient or phyto stimulant [28].

### Data analysis

Compost physico-chemical characteristics were analyzed using descriptive statistics and one-way analysis of variance on SPSS (version 22). The mean difference in physico-chemical characteristics between compost treatments were compared at 95% confidence interval level (p < 0.05). Simple correlation analysis was used to show the correlation of some physico-chemical characteristics of the compost.

## Results and discussion

### Physico-chemical characteristics of fresh MSW and organic additives

The moisture content of coffee pulp (59 ± 4.2%) is higher than leaves of *M. ferruginea* (54.9 ± 4.0%) and MSW (49 ± 3.4%) (Table 1). Raw composting materials moisture content (25–80%) is essential for successful organic material composting [29]. The bulk density of the MSW (0.28 ± 0.004 g/cm^3^) is higher than *M. ferruginea* (0.17 ± 0.005 g/cm^3^) and coffee pulp (0.13 ± 0.003 g/cm^3^). The EC of the MSW (4.1 ± 0.5mS/cm) is higher than coffee pulp (4.0 ± 0.4 mS/cm) and leaves of *M. ferruginea* (3.9 ± 0.4 mS/cm) (Table 1).

**Table 1:** Physico-chemical characteristics of fresh compost material

Types of raw material	Physico-chemical characteristics of the fresh composting materials							
	MC (%)	BD (g/cm^3^)	pH	EC (mS/cm)	OM (%)	TN (%)	P (ppm)	C: N
MSW	49 ± 3.4	0.28 ± 0.004	6.9 ± 0.8	4.1 ± 0.5	76.7 ± 3.5	1.7 ± 0.2	992 ± 10	26.2 ± 1.6
Coffee pulp	59 ± 4.2	0.13 ± 0.003	8.9 ± 1.4	4.0 ± 0.4	88.6 ± 2.4	2.7 ± 0.2	1360 ± 15	19.0 ± 2.3
*Millettia ferruginea* L.	54.9 ± 4.0	0.17 ± 0.005	6.2 ± 1.0	3.9 ± 0.4	81.4 ± 4.5	3.9 ± 0.3	1860 ± 13	12.1 ± 1.3
Forest soil	25.8 + 1.9	0.45 + 0.005	6.0 + 1.3	0.23 + 0.05	6.6 + 1.2	0.3 + 0.03	88.2 + 2.1	12.8 + 1.0
Top soil for pot media	13.5 + 2.1	0.56 + 0.004	5.0 + 1.4	0.18 + 0.05	4.1 + 0.9	0.19 + 0.05	45 + 1.9	12.5 + 1.3

*MC* moisture content, *BD* bulky density, *EC (mS/cm)*, *OM* organic matter, *TN* total nitrogen, *P* phosphorous, *C:N* carbon to nitrogen ratio

The pH of coffee pulp (8.9 ± 1.4) is higher than MSW (6.9 ± 0.8) and leaves of *M. ferruginea* (6.2 ± 1.0) (Table 1). The organic matter of coffee pulp (88.6 ± 2.4%) is higher than MSW (76.7 ± 3.5%) and leaves of *M. ferruginea* (81.4 ± 4.5%). The total nitrogen content of the leaves of *M. ferruginea* (3.9 ± 0.3%) is higher than coffee pulp (2.7 ± 0.2%) and MSW (1.7 ± 0.2%). The phosphorus content of *M. ferruginea* (1860 ± 13 ppm) is higher than coffee pulp (1360 ± 15 ppm) and MSW (992 ± 10 ppm). The C:N ratio of MSW (26.2 ± 1.6) higher than coffee pulp (19.0 ± 2.3) and *M. ferruginea* (12.1 ± 1.3) (Table 1).

### Physical characteristics of the compost treatments

#### Temperature

**Fig. 3:**
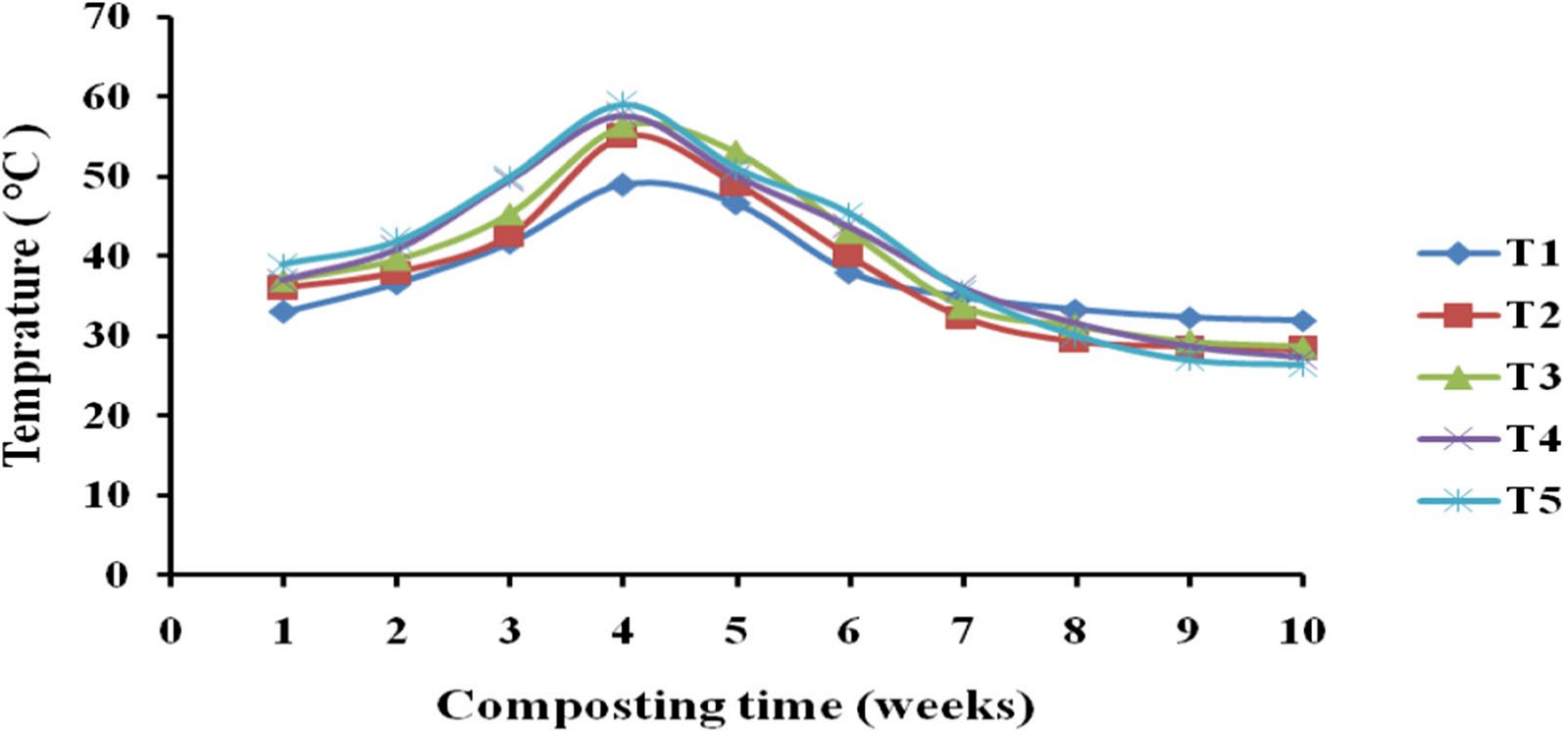
Temperature of treatments at different composting time

As illustrated in Table 2, the highest compost temperature was recorded in T1 (32.0 ± 1.0 °C), followed by T3 (28.7 ± 0.6 °C), and T2 (28.3 ± 1.2 °C). The temperature of T1 (MSW compost) was significantly different (*p* < 0.01) from all other treatments (MSW with different organic additives). The presence of high temperature on the compost in T1 may be due to the presence of microbial degradation of organic substrate in the compost heap under T1, as suggested by high C: N ratio compared to other compost treatments. The lower compost temperature was recorded in T5 (26.4 ± 1.0 °C). Similarly, a research reported that temperature fluctuation in the compost pile overtime related to consumption of organics by microorganisms [30]. In general, in all treatments an increase in temperature was observed at the early weeks (2–4 weeks) and a temperature decline at later weeks (5–10 weeks) of the composting period. An increase in compost temperature on the early weeks in all treatment shows an immediate commencement of microbial activities associated with respiratory metabolism in the compost heaps (Fig. 3).

**Table 2:** The physical characteristics of compost treatments (MSW alone & with organic additives)

Treatments	Physical characteristics of the compost treatments			
	T (°C)	MC (%)	BD (g/cm^3^)	EC (mS/cm)
T1 (control)	32.0 ± 1.0a	37.0 ± 1.1b	0.61 ± 0.02c	3.3 ± 0.3b
T2	28.3 ± 1.2b	45.1 ± 1.1a	0.45 ± 0.03b	4.4 ± 0.2a
T3	28.7 ± 0.6b	44.7 ± 0.6a	0.44 ± 0.02b	4.3 ± 0.2a
T4	27.3 ± 0.6bc	45.0 ± 0.9a	0.43 ± 0.03b	4.5 ± 0.3a
T5	26.4 ± 1.0c	45.5 ± 1.3a	0.36 ± 0.04a	4.6 ± 0.2a
Overall mean	28.6 ± 2.1	43.4 ± 3.4	0.48 ± 0.1	4.2 ± 0.5

*T* temperature, *MC* moisture content, *BD* bulk density, *EC* electrical conductivity

Mean values with different letters (a, b, c) among the treatments are significantly different from each other (*p* < 0.05)

However, a temperature more than 50 °C was recorded in MSW compost with various organic additives (T2, T3, T4 and T5). This suggests that the addition of various organic additives play a significant role in providing optimal C/N ratio to microorganism, and this encourage active microbial biomass, organic matter degradation and rise in temperature (Fig. 3). Similarly, another study reported that the presence of wide variety of organic additives within compost increase the number of microorganism and thus enhance cellulose degradation during composting of green organic wastes [31]. A research reported a decrease in temperature in MSW and sewage sludge compost as the composting process proceeds [32].

#### Moisture content

The highest moisture content was recorded in T5 (45.5 ± 1.3%), followed by T2 (45.1 ± 1.1%) and T4 (45.0 ± 0.9%) (Table 2). There was a significant difference (*p* < 0.05) in moisture content between T5 and T1, T2 and T1, T4 and T1, T3 and T1. There was no significant difference between T5 and other treatments of T2, T3 and T4. The presence of high moisture content on treatment amended with different organic additives (T2, T3, T4 and T5) may be associated with the presence of relatively high organic matter and low temperature compared to T1. Likewise, other study suggested that organic matter content of the compost increases water holding capacity and ideal moisture content [33]. Similarly, study report showed that moisture content of the compost is affected by the temperature and microbial activity [34]. A research report also showed that 29–48% moisture content on the final compost [15]. The moisture content of all the treatments except T1 was within the compost quality standard set by Ontario Ministry of the Environment (40–55%) [35] and Ethiopian Federal Environmental Protection Authority (EFEPA) (40–60%) [36]. However, moisture content of all the treatments are greater than the moisture content (23–32%) of matured compost [23] and less than the moisture content (54–59%) of matured compost [12].

#### Bulk density

**Fig. 4:**
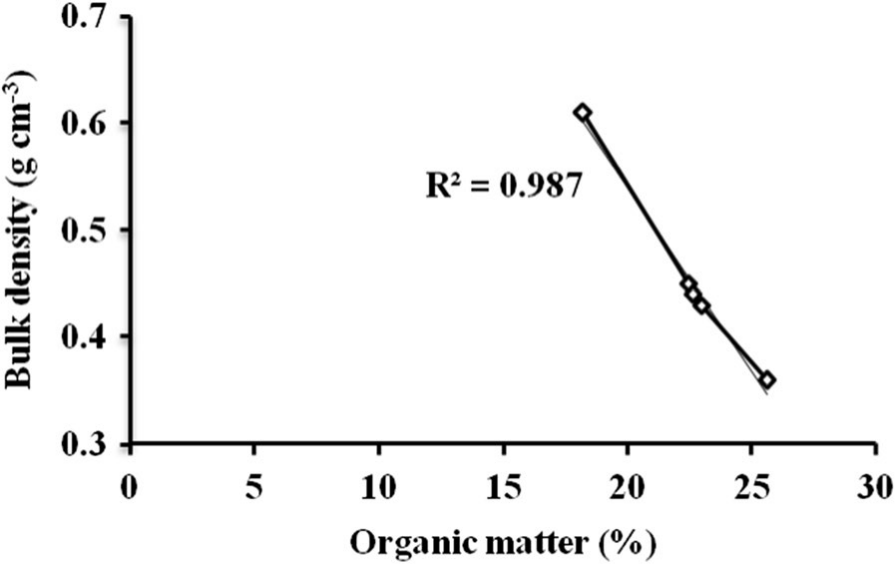
The relationship between bulk density and organic matter

As indicated in Table 2, the highest bulky density was recorded at T1 (0.61 ± 0.02 gm/cm^3^), and lowest was recorded at T5 (0.36 ± 0.04 gm/cm^3^). The compost bulk density in T1 is significantly different (*p* < 0.05) from T2, T3, T4 and T5. The presence of high bulk density in T1 may be related with the presence of low organic matter in T1. The presence of organic additives like coffee pulp, coffee effluent and leaves of *M. ferruginea* increased compost organic matter, in turn decreased the bulk density of the compost treatments with organic additives. More importantly, the strong negative correlation (R^2^ = 0.987) between the bulk density and the organic matter content of the treatment shows the role of additive on the bulk density (Fig. 4). Similarly, another study reported that bulk density of the compost increases with decreasing the compost organic matter and vice-versa [23, 37]. The present study finding of bulk density is in-line with bulk density (0.42–0.65 gm/cm^3^) [23], and (0.46–0.56 gm/cm^3^ [38]) of matured compost.

#### Electrical conductivity (EC)

As shown in Table 2, the highest electrical conductivity was recorded in T5 (4.6 ± 0.2 mS/cm) followed by T4 (4.5 ± 0.3 mS/cm) and the lowest was recorded in T1 (3.3 ± 0.3 mS/cm). The EC of the compost in T5 is significantly different from T1. However, no significant difference was recorded in electrical conductivity of the compost between T5 and other treatments (T2, T3 and T4). The presence of high EC in those treatments (T5, T4, T3 and T2) may be related with the released of base cation (K^+^, Ca^2+^, Na^+^) and inorganic salts (phosphate and ammonium ions) during microbial decomposition of added organic matter. In general, the reported EC is in-line with the EC content (1.5–8.8 mS/cm) [16], 4.2–5.2 mS/cm [38] and 2.2–9.3 mS/cm [39] of matured compost. However, the EC content of the compost treatments are greater than the EC content (1.9–2.7 mS/cm) of the matured compost [12].

### Chemical characteristics

#### pH

As indicated in Table 3, the higher pH of the compost was recorded in T5 (7.9 ± 0.1) and T4 (7.9 ± 0.1). There was significant difference (*p* < 0.05) in between T5 and T1, T5 and T2; and T5 and T3. However, no significant difference was recorded in pH of the compost between T5 and T4. The presence of high pH in T5 and T4 may be related with the release of base cations like, K^+^, Ca^+^, Mg^2+^ and Na^+^ during microbial biodegradation of various organic additives like coffee pulp, coffee effluent and *M. ferruginea*. Similarly, a study report showed that the increase in pH of the compost is attributed to the releases of base cation during decomposition of various organic additives [19]. The pH of the compost treatments are above the pH (6.9–7) of the matured compost [38], and below the pH (8.3–8.4) of the matured compost [12]. Further, this study finding is in agreement with the quality compost pH standard of Republic of South Africa (5–8), Switzerland (< 8.2) and UK (7–8.7) [40–42] and pH (6.3–7.8) of matured compost [23].

**Table 3:** Physico-chemical characterization of compost treatments (MSW alone and integrated with organic additives) at 70 days

Chemical Characteristics	Compost treatments					
	T1 (control)	T2	T3	T4	T5	Over all mean
pH	7.1 ± 0.1c	7.6 ± 0.1b	7.6 ± 0.1b	7.9 ± 0.1a	7.9 ± 0.1a	7.6 ± 0.3
OM (%)	18.2 ± 0.7c	22.5 ± .0.8b	22.6 ± 0.9b	23 ± 0.9b	25.6 ± 0.8a	22.4 ± 2.5
OC (%)	10.6 ± 0.4c	13.0 ± 0.5b	13.1 ± 0.5b	13.3 ± 0.5b	14.9 ± 0.5a	13 ± 1.5
TN (%)	0.74 ± 0.05c	1.0 ± .0.06b	1.0 ± 0.04b	1.2 ± 0.05a	1.2 ± 0.05a	1.0 ± 0.2
T/P (ppm)	2051 ± 141c	2542 ± 122b	2576 ± 142b	2834 ± 151a	2918 ± 114a	2584 ± 315
C: N	14.3 ± 0.4a	13.0 ± 0.6b	13.0 ± 0.8b	11.3 ± 0.3c	12 ± 0.4c	12.7 ± 1.1

*OM* organic matter, *OC* organic carbon, *TN* total nitrogen, *T/P* total phosphorus, *C:N* carbon and nitrogen ration

* Mean values with different letters (a, b, c) among the treatments are significantly different from each other (*p* < 0.05)

#### Organic matter (OM)

The highest organic matter content of the compost was recorded in T5 (25.6 ± 0.8%) (Table 3). There was a significant difference (*p* < 0.05) between T5 and T4, T5 and T3, T5 and T2 and T5 and T1. Similarly, organic matter content of T4, T3 and T2 are significantly different from T1. The presence of high organic matter in T5 may be related with the presence of organic additives like coffee pulp and *M. ferruginea*. Also, the presence of relatively high organic matter in T2, T3 and T4 associated with the presence of additives like coffee effluent, coffee pulp and *M. ferruginea* respectively*.* The organic matter content of these treatments was in line with the organic matter content of German compost quality standards (> 15%), Austrian (> 20%) and South Africa (20–50%) and organic matter (17–22%) of matured compost [15]. However, except T5, the organic matter content of all treatments have less organic matter content compared to organic matter reported by UK ≥ 25% [43] and Ethiopia ≥ 25% [36] and organic matter (28–42%) of the matured compost [23, 38].

#### Total nitrogen (TN)

The highest total nitrogen content was recorded in T4 (1.2 ± 0.05%) and T5 (1.2 ± 0.05%) (Table 3). There was significant difference in total nitrogen content between T5 and T3, T5 and T2, and T5 and T1. Similarly, there was significant difference (*p* < 0.05) between T4 and T3, T4 and T2; and T4 and T1. The presence of high total nitrogen in T5 and T4 may be related with the presence of *M. ferruginea* in those treatments. This study result is in-line with the research reported an increases in nitrogen content in soil supplemented with leaves of *M. ferruginea* [44]. The nitrogen content of the matured compost of all the treatments in agreement with the nitrogen content of Netherland, Belgium and Italy compost quality standard (> 0.7%) and the range reported by Switzerland agriculture (1.05–1.13%) [41, 45].

#### Total phosphorous (P)

Table 3 illustrated that the highest available phosphorous content was recorded in T5 (2918 ± 114 ppm). There was significant difference (*p* < 0.05) in available phosphorus content between T5 and T1, T5 and T2, T5 and T3. The presence of high available phosphorus in T5 and T4 may be related with the releases of available phosphorus during microbial degradation of organic additives like coffee pulp and *M. ferruginea*. Similarly, a research study reported that leaves litter of *M. ferruginea* and coffee pulp increases available phosphorous content of the soil [12, 46]. Further, another study stated that the difference in available phosphorus content is related with the difference in raw material used for composting [16]. The finding is in-line with the available phosphorous content (1610–2240 ppm) of matured compost [47].

#### C: N ratio

The highest C:N ratio was noted in T1 (14.3 ± 0.4), while the lowest was recorded in T4 (11.3 ± 0.3) (Table 3). There was significant difference (*p* < 0.05) in C:N ratio between T1 and T2, T1 and T3, T1 and T4; and T1 and T5. The presence of high C:N ratio in T1 may be related with low microbial degradation because of imbalance in C:N ratio on composting material. The C:N ratio of all the treatments were in agreement with the C:N ratio (< 25:1) of stable compost [48]. Similarly, the C:N ratio of the final compost is in-line with C:N ratio (10–15), and a manifestation of an advanced process of composting and a good retention of the nitrogen content which favors the conservation of nitrogen as nutrient [49]. Similarly, the finding of this C:N ratio is above C:N (9–11) of the matured compost [15]; but in agreement with the C:N ratio reported by Ethiopian Federal Environmental Protection Authority (EFEPA) (< 29:1) and European Union (EU) and Composting Council of Canada (< 25:1) [36].

#### Phytotoxicity of compost

Germination index is considered the most sensitive parameter for identifying the phytotoxicity of the compost and assess its suitability for use as soil amendment or growing media. As mentioned in Table 4, based on the soil and compost composite phytotoxic test (3:1 ratio), the highest germination index (GI) was recorded on T5 (137%), followed by T4 (133%), T3 (133%), T2 (118%) and T1 (107%). This shows that germination index of all treatments are above 100% (GI > 100%), and thus all the treatments are important as phytonutrient or phyto-stimulant for the lettuce plant. Similarly, a research reported that cucumber seed germination index 80–160% in various municipal solid waste and organic additives compost media [50].

**Table 4:** The Phytotoxicity of the treatments

Treatments	Phytotoxicity of compost				
	Soil and compost (3:1)		Soil and compost (0:1)		Remark
	GI (%)	GI index	GI (%)	GI index	Standard GI values:
T1	107	Phytonutrient	72	Moderately	Highly phytotoxic: < 50%
T2	118	Phytonutrient	82	Non-phytotoxic	Moderately phytotoxic: 50–80%
T3	133	Phytonutrient	82	Non-phytotoxic	Non-phytotoxic: > 80%
T4	133	Phytonutrient	101	Phytonutrient	Phytonutrient: > 100%
T5	137	Phytonutrient	102	Phytonutrient	(Zucconia, 1985)

However, the phytotoxic test using compost (100%) showed that the high germination index was recorded on T5 (102%), followed by T4 (101%), T3 (82%), T2 (82%) and T1 (72%) (Table 4). This indicates that the germination index of T5 and T4 are greater than 100% and are phytonutrient for the lettuce plant. Since, the germination index of T3 and T2 are greater than 80%, they are non-phytotoxic to lettuce. However, the germination index of T1 is in between 50–80%, it is moderately phototoxic to lettuce plant. The low GI in T1 could be due to the existence of phenol acids and volatile fatty acids on T1. A study reported that the presence of acetic acid, phenolic compounds, ethylene oxide and ammonia content hinder seed germination and plant root growth [51]. Also, another study reported 60% germination index in kitchen waste compost [52].

In general, the germination index of the treatments showed that all the treatments compost and soil composite media (3:1, soil and compost) do not have phytotoxicity effect and are an important phytonutrient for lettuce plant. However, only T5 and T4 are an important phytonutrient for lettuce plant in 100% compost media.

## Conclusions and recommendations

Municipal solid waste (MSW) composting with other locally available organic additives like coffee pulp, coffee effluent and *M. ferruginea* separately and in combination (MSW + coffee pulp + *M. ferruginea*) is important for compost quality amelioration and production of phytotoxic free compost. Most importantly, T4 (MSW + *M. ferruginea*) and T5 (MSW + coffee pulp + *M. ferruginea*) have higher compost quality and are important phytonutrient for lettuce plant. Amending soil media with compost treatments in 3:1 is important for lettuce seed germination, root elongation and overall soil media nutrient amendment. Since, southwest Ethiopia soil is acidic in nature, the presence of high EC on the compost treatments are important for amelioration of soil acidity problem in the region.

Thus, for effectively management of the 75% of organic fraction of waste generated from households in the study area, recycling methods like composting with organic additives must be used at large. Hence, municipality must work towards waste recycling and composting as waste reduction measures. Therefore, municipality must create awareness to society regarding separation of organic waste from altogether, and recycling of waste by composting.

## Supplementary Information

**Additional file 1:** Field experimental photos of composting treatment

**Additional file 2:** Compost dataset and analysis

## Data Availability

All data generated or analyzed during this study are included in this present article and in additional information files.

## Abbreviations

MSW: Municipal solid waste
EC: Electrical conductivity
OC: Organic carbon
TN: Total nitrogen
CRD: Complete randomized design
GI: Germination index
MC: Moisture content
BD: Bulk density
OM: Organic matter
T1: Treatment 1
T2: Treatment 2
T3: Treatment 3
T4: Treatment 4
T5: Treatment 5
EFEPA: Ethiopian Federal Environmental Protection Authority
EU: European Union
