# Supplementary figures and images for: Evaluation of compost quality from municipal solid waste integrated with organic additive in Mizan–Aman town, Southwest Ethiopia
Source: BMC Chem. 2021 Jul 19;15(1):43. doi: 10.1186/s13065-021-00770-1 (PMC8290552; doi:10.1186/s13065-021-00770-1)

| 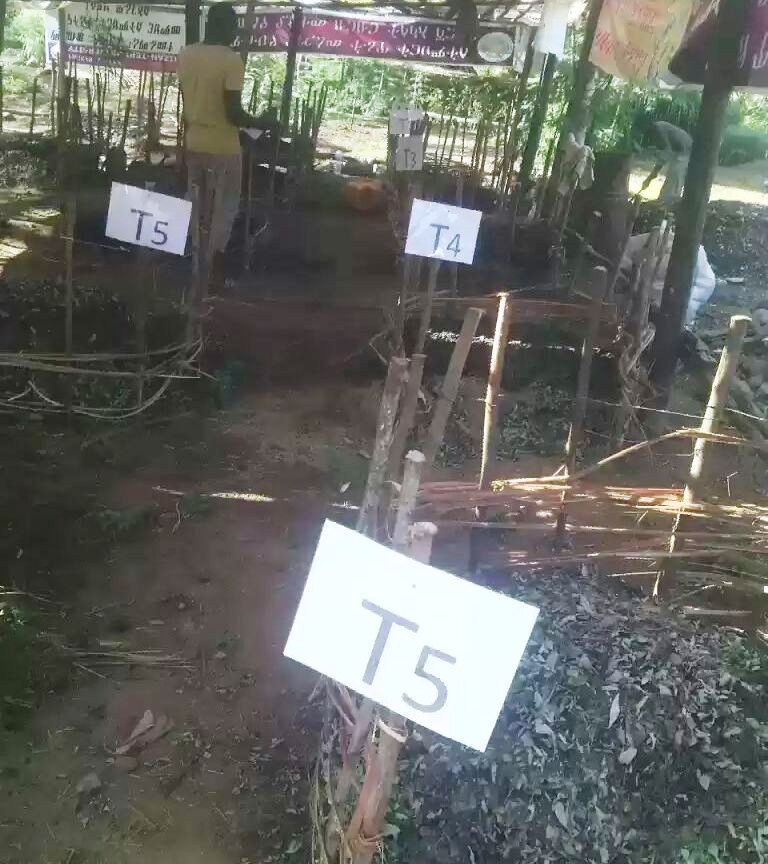 | 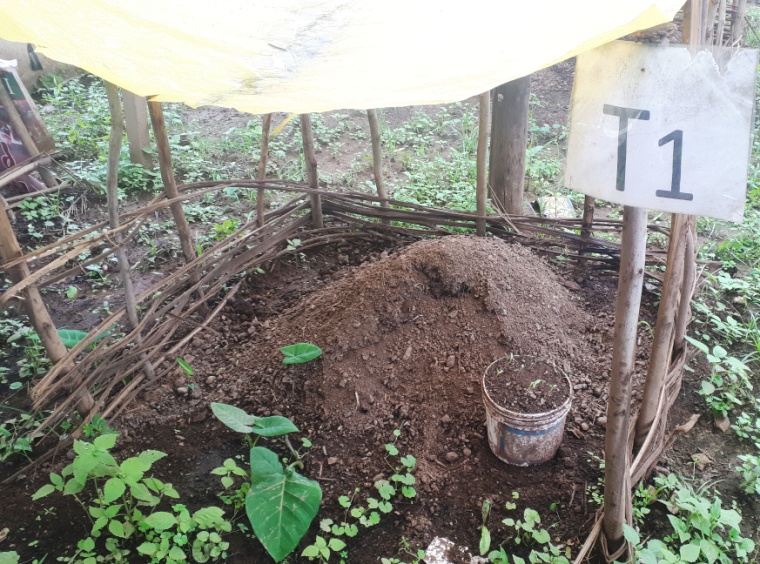 |
| --- | --- |
| 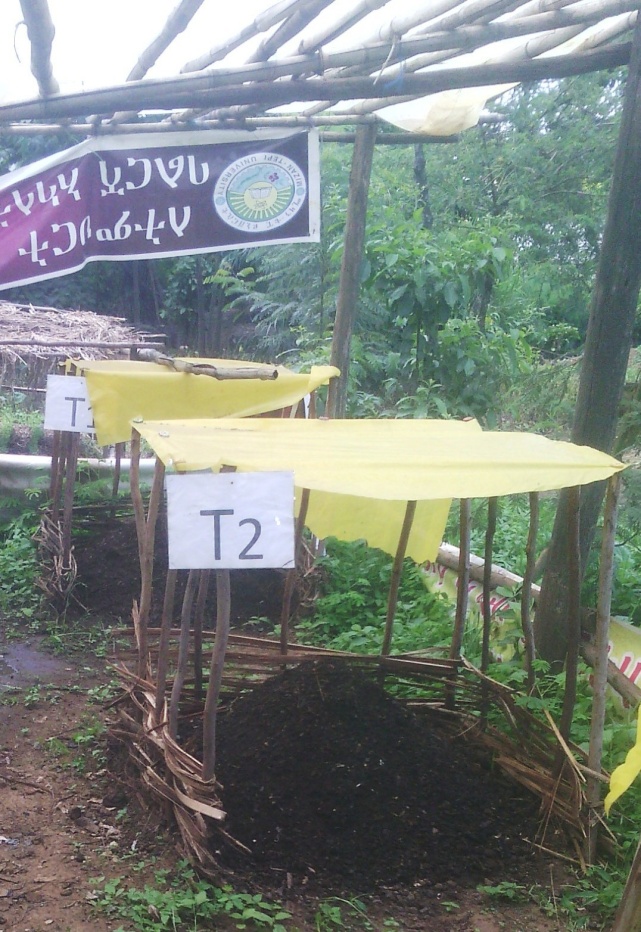 | 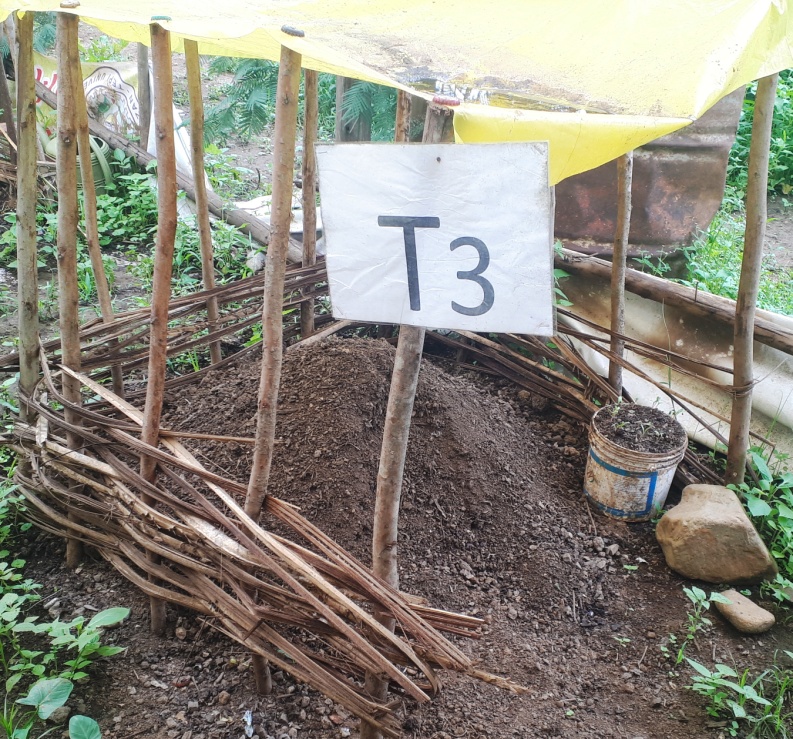 |
| 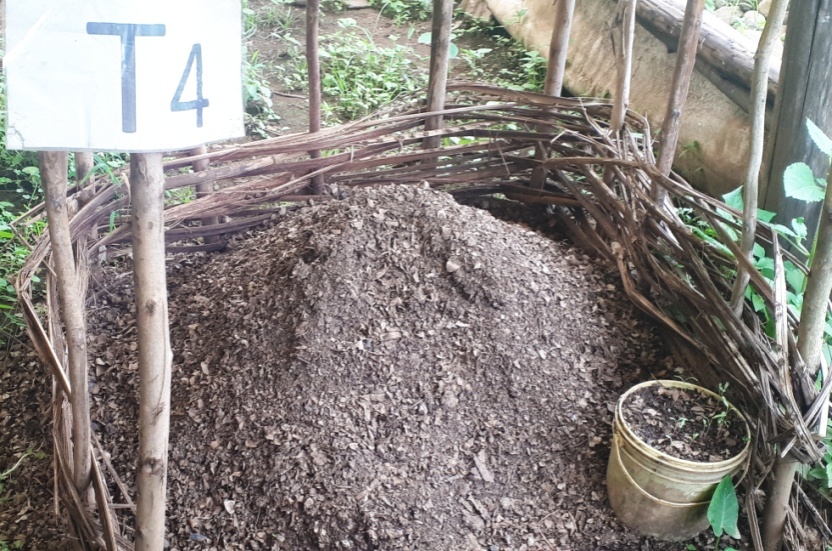 | 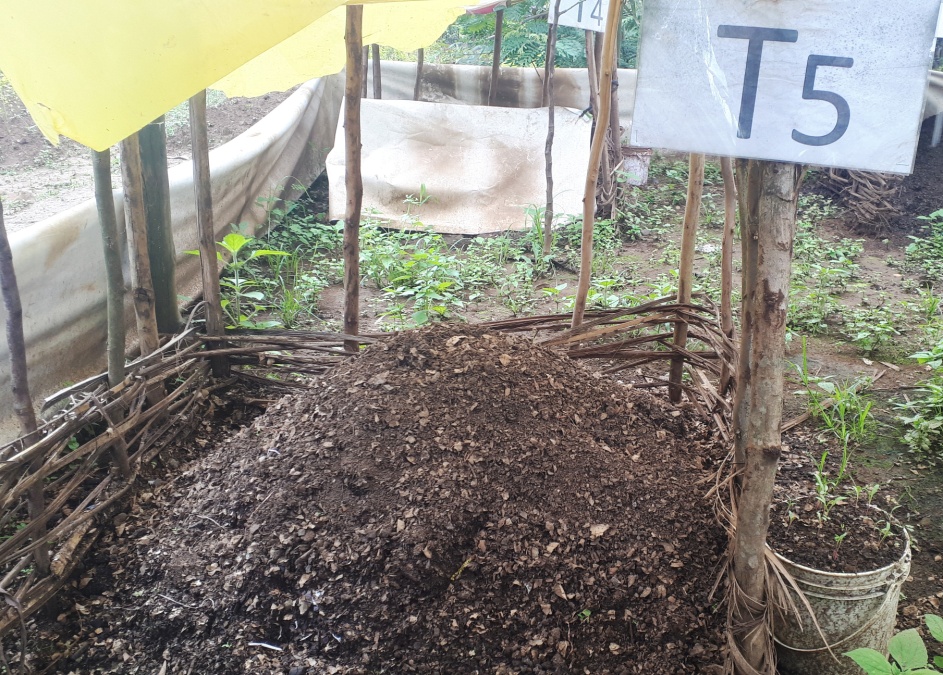 |

Supplement: Supplementary file 1 — Additional file 1: Field experimental photos of composting treatment [file 13065_2021_770_MOESM1_ESM.docx]
